# Blood pressure variability compromises vascular function in middle-aged mice

**DOI:** 10.1101/2024.10.21.619509

**Published:** 2024-10-24

**Authors:** Perenkita J. Mendiola, Philip O’Herron, Kun Xie, Michael W. Brands, Weston Bush, Rachel E. Patterson, Valeria Di Stefano, Jessica A. Filosa

**Affiliations:** 1 Department of Physiology, Medical College of Georgia, Augusta University, Augusta, Georgia, USA

## Abstract

Blood pressure variability (BPV) has emerged as a novel risk factor for cognitive decline and dementia, independent of alterations in average blood pressure (BP). However, the underlying consequences of large BP fluctuations on the neurovascular complex are unknown. We developed a novel mouse model of BPV in middle-aged mice based on intermittent Angiotensin II infusions. Using radio telemetry, we demonstrated that the 24-hr BP averages of these mice were similar to controls, indicating BPV without hypertension. Chronic (20–25 days) BPV led to a blunted bradycardic response and cognitive deficits. Two-photon imaging of parenchymal arterioles showed enhanced pressure-evoked constrictions (myogenic response) in BPV mice. Sensory stimulus-evoked dilations (neurovascular coupling) were greater at higher BP levels in control mice, but this pressure-dependence was lost in BPV mice. Our findings support the notion that large BP variations impair vascular function at the neurovascular complex and contribute to cognitive decline.

## INTRODUCTION

Dementia, the 7^th^ leading global cause of mortality, presents as the predominant neurodegenerative complication in the elderly population. Notably, hypertension emerges as a primary risk factor for dementia^1, 2^. Blood pressure (BP) regulation involves a complex interplay of central and peripheral mechanisms, finely attuned to acute and chronic stressors in maintaining physiological ranges. Perturbations in vascular function and structure (e.g., reactivity, vascular stiffness^3^), alterations in baroreflex sensitivity^4^, and enhanced sympathetic activation^5^ are recognized factors that disrupt BP balance. Blood pressure variability (BPV) (an index of BP *fluctuation or variation*), particularly in midlife, increases the risk for cognitive decline^6^, end-organ damage^7^, and cardiovascular events^7, 8^. Increased BPV also serves as an early predictor of hypertension^9^. BPV delineates fluctuations across various measurement periods—from very short-term (beat-to-beat) to long-term (months or years^10^).

Given the evidence that elevated BPV can precede hypertension^11^, a modifiable risk factor in the setting of vascular cognitive impairment and dementia; understanding the drivers and cellular targets of BPV is essential. Unlike hypertension, BP fluctuations often elude detection in screening practices conducted in clinical settings, which rely on singular or averaged BP measurements^12^. The absence of a gold standard for BPV indexing^13^ and limited insights into the magnitude and frequencies of impactful BP fluctuations in disease conditions further compound this issue. Additionally, the absence of suitable animal models has limited the understanding of how this overlooked variable may impact brain health. In this study, we sought to establish an innovative murine model of BPV and to interrogate the impact of large BP fluctuations on cardiovascular and neurovascular outcomes.

Cerebral blood flow (CBF) is tightly regulated by diverse signaling processes, including the interaction of cellular elements comprising the neurovascular complex—endothelial cells, vascular smooth muscle cells, pericytes, astrocytes, neurons, and microglia^14, 15^. Mechanisms governing CBF operate under both steady-state conditions (e.g., cerebral autoregulation (CA), humoral processes, chemoregulation) and in response to local neuronal activity (e.g., neurovascular coupling (NVC))^16^. Evidence suggests that sustained hypertension adversely affects the functional integrity of the neurovascular complex^17–19^, compromises cerebral perfusion^17, 18^ and contributes to cognitive decline^18^. However, the impact of large BP fluctuations on steady-state or activity-evoked CBF changes remains poorly understood.

Chronic hypertension leads to adaptive processes (e.g., vascular remodeling^20, 21^) and a rightward shift in the cerebral autoregulation (CA) curve^22^, heightening vulnerability to ischemia at low BP while shielding the brain from hyperperfusion at high pressure. In humans, hypertension and BPV have been associated with white matter hyperintensities^23, 24^ and microbleeds^25–27^. Intriguingly, the impact of BPV (prior to hypertension onset) on cerebrovascular function and neurovascular outputs has received little attention.

Using an innovative murine model of BPV, in the absence of overt hypertension, combined with *in vivo* two-photon imaging, we show that chronic BP fluctuations lead to microvascular dysfunction (e.g., enhanced parenchymal arteriole myogenic responses) and a blunted NVC response. In addition, mice subjected to chronic BPV showed poor cognitive performance. These findings underscore the pivotal role of dysregulated BP events in brain health and function.

## METHODS

### Animals

All experiments were conducted in middle-aged (12–15 months old) male C57BL6 mice (Jackson Laboratories) following protocols approved by the animal care and use committee of Augusta University (AU). Before experimentation, animals were housed in a room maintained at 20–22°C with a 12hr:12hr light-dark cycle and given *ad libitum* access to food and water.

### Craniotomy surgery for chronic window

Surgeries were conducted using the aseptic technique. Animals were injected with dexamethasone and meloxicam 2–4 hours before surgery to prevent edema and/or inflammation. General anesthesia was induced with isoflurane (2%) and following loss of reflex, hair was removed from the scalp and the mouse was transferred to a sterile field. A single injection of ketamine/dexdomitor (60 mg/kg/0.5mg/kg) was administered to maintain anesthesia in the sterile field. The scalp was scrubbed with betadine/alcohol (3 times each, alternating). A scalp incision was made (~1 cm) and a small craniotomy opened over the visual cortex (1–2 mm). A small aluminum head holder (300 mg) was fixed to the skull using cyanoacrylate glue followed by dental cement. The bone flap was removed (~3 mm diameter). A glass coverslip (3 mm) was glued to a 4 mm coverslip and placed into the craniotomy with the inner glass (3mm) touching the cortex. The edges of the coverslip were then sealed with cyanoacrylate glue followed by dental cement covering everything including the wound margins.

### Telemetry and iPrecio pump implantation surgery

Following a 3-week recovery period after craniotomy surgery, mice were implanted with a programmable pump (iPrecio, SMP-310R) and a biotelemetry transmitter device (PA-C10, Data Science International). Mice underwent brief anesthesia in a small chamber using 5% isoflurane for induction, followed by maintenance of anesthesia via a nose mask with 1.5–2% isoflurane. The neck fur was carefully clipped on both the anterior and posterior sides. Subsequently, mice were placed in a sterile field in a ventrally recumbent position, and a 1.5 to 2 cm horizontal incision was made. The pump and biotelemetry transmitter device were then implanted subcutaneously. The pump catheter was trimmed to 0.5 cm for subcutaneous drug delivery, while the telemeter catheter was tunneled subcutaneously over the shoulder, with its tip positioned within the left carotid artery. Closure of incisions was performed using 5–0 sutures, after which the mice were gradually brought out of anesthesia. It is important to note that due to the complexity of the surgeries involved, mice used for behavioral studies were not implanted with a telemetry catheter or cranial window. However, these mice were individually housed to simulate the conditions of the telemetry cohort.

### Blood pressure assessment and BPV induction

After recovery from surgery, mice were housed individually in standard mouse cages under the conditions described above and assigned to a control or experimental BPV group. During this recovery period (~7 days), both groups were infused with saline to determine baseline cardiovascular parameters. Blood pressure signals encompassing mean arterial pressure (MAP), systolic blood pressure (SBP), diastolic blood pressure (DBP), heart rate (HR), and pulse pressure (PP) (determined from the difference between the SBP and DBP) were continuously sampled at 250 Hz for 10 seconds and collected every 30 seconds for 24 hours per day. Once a baseline MAP was established, the saline in the infusion pump (in the BPV group) was substituted with angiotensin II (Ang II, Sigma Aldrich, A9525) and administered intermittently at a calculated dose of 3.1 μg/hour (every 3–4 hours) for 25 days.

### Two-photon imaging

On the day of the two-photon imaging acquisition, a mouse was anesthetized with chlorprothixene (0.04 cc) and a low dose of isoflurane (≤0.8%); a protocol to mildly sedate mice^28^. An intra-orbital injection of Texas red (5%, 40 μl) was administered to label blood vessels. The mouse was head-fixed and a picospritzer (positioned on the contralateral hemisphere intended for imaging) was used to deliver a puff of air for whisker stimulation (WS) at a rate of 10 Hz for 20 seconds repeated seven to eight times with ~ 90-second delay between each WS during an imaging session. Imaging sessions were conducted 106 ± 3.6 μm below the brain’s surface while pump infusion was *off* (low BP period) and again during Ang II infusions (high BP period). Images were collected at a rate of 3.75 frames/second.

### Behavioral studies

Behavioral tasks were performed by an experimenter at the AU small animal behavioral core (SABC), who was blinded to the experimental protocol. BPV mice were subjected to behavioral tests at two-time points. First during the saline infusion period (days 7–10), with observations considered as baseline, and second, 25–26 days post-Ang II infusions. About 30 minutes before training and testing, animals were brought to the testing room to acclimate. Animals remained in the laboratory for 15 minutes following study completion. To eliminate olfactory cues, animal droppings were removed, and the space and objects were cleaned (dilute 50% (vol/vol) ethanol solution) between sessions where appropriate.

#### Novel object recognition (NOR) task.

Animal behavior was assessed using the previously described NOR task procedure^29^. Animals were acclimated and then familiarized with an opaque plastic chamber (78.7 cm × 39.4 cm × 31.7 cm) containing bedding for 10 minutes. The next day, a training session involved animals exploring two identical objects for 10 minutes before returning to their home cages. Object recognition was assessed one hour later by placing an animal in the NOR apparatus with a familiar object and a new or novel object for 10 minutes. The objects’ positions and roles (familiar or novel) were randomly assigned. Objects were positioned about 40 cm apart, 19.3 cm from the two short walls and 19.3 cm from the two long walls of the chamber. Exploration of an object was defined as direct interaction with nostrils or head position towards the object at a distance ≤ 2 cm. For data inclusion, a mouse had to explore an object for at least 4 seconds and spend a minimum of 10 seconds of total object exploration. Recognition index was calculated using the formula: $\text{Recognition}\;\text{Index}=\frac{\left(\text{time}\;\text{spent}\;\text{at}\;\text{novel}\;\text{object}\right)}{\left(\text{time}\;\text{spent}\;\text{at}\;\text{novel}+\text{familiar}\;\text{objects}\right)}$. The discrimination index was calculated as: $\text{Discrimination}\;\text{Ratio}=\frac{\left(\text{time}\;\text{spent}\;\text{at}\;\text{novel}-\text{familiar}\;\text{object}\right)}{\left(\text{time}\;\text{spent}\;\text{at}\;\text{novel}+\text{familiar}\;\text{objects}\right)}$. The cohort’s recognition capacity was accessed prior to testing at one, four, and 24-hour delaysThe one-hour delay for testing was determined based on earlier NOR tests assessing the cohort’s recognition capacity at one, four, and 24-hour delays. The animals exhibited normal recognition capacity with a one-hour delay but failed at 4- and 24-hour delays. Hence, the one-hour delay was selected for subsequent tests.

#### Y-maze.

Y-maze tasks were performed twenty-four hours after NOR tasks. The Y-maze consisted of three arms (35.4 cm long, 9.9 cm wide with a height of 13.8 cm). Spontaneous alternation behavior was assessed by randomly placing mice in one of the three arms and allowed to explore for 10 minutes. Arm entries were visually scored into a series where a triplet set of arm entries constituted an alternation. An alternation was defined as successive consecutive entries into three different arms, and maximum alternations as the total number of arm entries minus 2. Percent alternation was calculated as the proportion of true alternations out of maximum alternations (# of true alternations/# of maximum alternations) × 100).

### Data analysis.

Two-day averaged values of 24-hour or 12-hour (active and inactive period) BP variables were compared to a baseline phase corresponding to saline infusion (~ 5 days). BPV was calculated using the average real variability (ARV) index $\left(\text{ARV}=\frac{1}{\text{N}-1}\sum_{\text{K}=1}^{\text{N}-1}\text{x}\left|{\text{BP}}_{\text{K}+1}-{\text{BP}}_{\text{K}}\right|\right)$ where K is the order of measurements and n denotes the number of BP readings. Absolute BP values were extracted every 15 minutes and averaged for the hour. The coefficient of variation (CV) was also used to calculate BPV. CV was calculated as the ratio of the standard deviation of hourly BP averages to the 24-hour BP average $\left(\text{CV}=\frac{\text{Standard}\;\text{Deviation}(\text{Hourly}\;\text{BP}\;\text{Average})}{24\text{Hour}\;\text{BP}\;\text{Average}}\right)$. Similar to BP variables, two-day averages of 24-hour and 12-hour ARV and CV were compared to the average value of the last 5 days during the baseline phase. The bradycardic gain was calculated using (Bradycardic Gain = (HR at high BP – HR at low BP)/(SBP at high BP – SBP at low BP)). Parenchymal arteriole diameter responses to changes in MAP (myogenic responses) and whisker stimulation (WS) were analyzed using MatLab and ImageJ^30, 31^. A linear regression was determined from the relationship between the MAP and arteriole diameter changes to evaluate myogenic responses. For neurovascular coupling experiments, the baseline was defined as 30 frames before stimulus onset. The stimulus-response comprised 100 frames encompassing the 20-second stimulus and the first 6 seconds post-stimulus. Six to eight WS events were averaged for each run and responses were normalized to the baseline average.

#### Exclusion Criteria:

We observed unexplained random transient dilatory events during two-photon imaging sessions, **Supplemental Figure 1 B, D**. These events occurred in control and BPV mice during low and high BP periods. Because these random dilations have been observed in other mouse strains using comparable *in vivo* imaging settings, they may be attributed to the sedative. To isolate pressure-evoked parenchymal arteriole changes (myogenic studies Figure 4), random dilatory events corresponding to baseline tone were removed from the analysis, **Supplemental Figure 1**.

GraphPad Prism 10 software (GraphPad Software, La Jolla, CA) was used for all statistical analyses. Values are expressed as mean ± S.E.M. A minimum of three mice was used for each experimental data set, and the specific sample size (n) is defined in figure legends. Data was tested for normal distribution, and statistical tests were used accordingly. Differences between two means within groups were determined using paired Student’s *t*-test. Differences between groups were determined using Student’s unpaired *t*-test, one- or two-way ANOVA with corresponding multiple comparison *post hoc* test specified in figure legends. Statistical significance was tested at a 95% (*P* < 0.05) confidence level denoted with the corresponding symbol in figure legends.

## RESULTS

### Pulsatile Ang II infusion-induced changes in blood pressure

**Figure 1.**
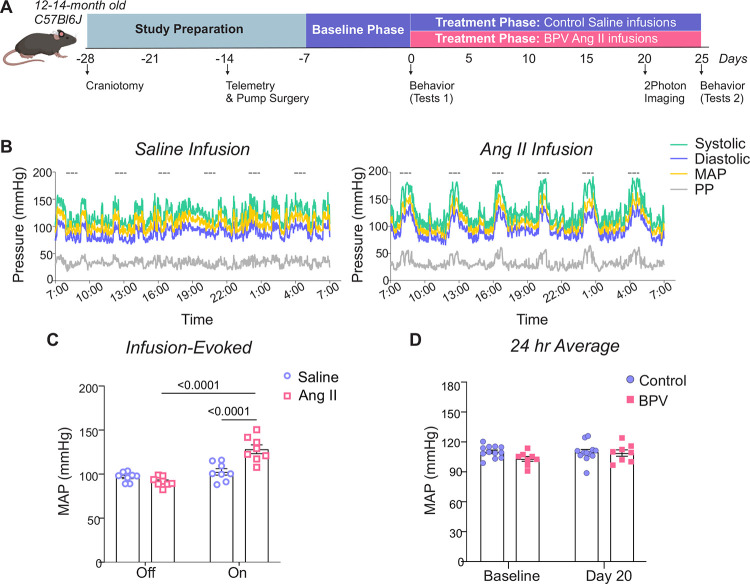
Experimental design and Ang II infusion effects on mean arterial pressure. **A**, Illustration of the experimental design used for middle-aged C57BL6J male mice including implantation of a chronic cranial window (−28 days from treatment onset) and an infusion pump and telemetry (−7 days from treatment onset). Pumps were programmed to infuse saline (baseline phase) or angiotensin II (Ang II) (treatment phase) in an intermittent manner, 1 hour every 3–4 hours or 18.4 μg/day. *In-vivo* 2P imaging sessions were conducted on ~ day 20 into the treatment phase. Behavioral tests were conducted during the baseline period (Test 1) and on 25 day into the treatment phase (Test 2). **B**, Representative raw traces of 24-hour MAP, SBP, DBP, and PP during intermittent saline (left) or Ang II infusions (right). Dashed lines correspond to the period (1 hr) when the pump is *on* (infusing). **C**, Average MAP (5 minutes) while the pump is *Off* or *On*. **D**, 24-hour average MAP during a 5-day saline infusion phase (Baseline) and on day 20 of treatment (Day 20). Two-way ANOVA repeated measures followed by Sidak’s multiple comparisons test (C, n = 8 BPV mice) (D, n = 12 control mice, n=8 BPV mice). **Ang II**=Angiotensin II, **BPV**=blood pressure variability, **MAP**=mean arterial pressure

We developed a novel murine model of high BPV using the experimental design shown in Figure 1A. The protocol consisted of a baseline phase (~5 days), corresponding to saline infusions in all mice, followed by a treatment phase (~20–25 days), where mice were either left on saline (control group) or subjected to robust BP transients induced via Ang II infusions (BPV group). Subcutaneous pumps were programmed to infuse 2 μL 6–8 times per day, with infusion periods corresponding to 1 hour every 3–4 hours (depending on the number of pulses), Figure 1B, right panel. During the pump-*off* periods, BP corresponded to 97±2.0 mmHg and 91±1.9 mmHg for MAP (Figure 1C), 113±2.6 mmHg and 105±2.6 mmHg for SBP, 80±1.7 mmHg and 78±1.7 mmHg for DBP, and 34±1.3 mmHg and 27±1.8 mmHg for PP, for mice with pumps infusing saline vs. Ang II, respectively, **Supplemental Figure 2A**. During the pump-*on* period, saline infusion did not change BP. However, Ang II infusions evoked significant BP transients corresponding to Δ37±3.9 mmHg (P<0.0001) for MAP (Figure 1C), Δ70±3.0 mmHg (P<0.0001) for SBP, Δ29±4.1 mmHg (P<0.0001) for DBP, and Δ28±3.0 mmHg (P<0.0001) for PP, **Supplemental Figure 2A**. Despite robust BP transients, 20 days of treatment minimally affected the 24-hour averages (vs Baseline) in all cardiovascular variables, Figure 1D & **Supplemental Figure 2B**. The delta BP (Baseline vs Day 20) for MAP was −0.46±2.0 mmHg and 6±2.4 mmHg for control and BPV mice, respectively, Figure 1D & **Supplemental Figure 2B**. While the SBP in BPV mice significantly increased Δ9±2.9 mmHg (P=0.0088), mice remained within the lower range of high BP (131±4.4 mmHg). Of note, the baseline DBP of BPV (vs control) was significantly lower (P=0.0192) before treatment, **Supplemental Figure 2B**.

**Figure 2.**
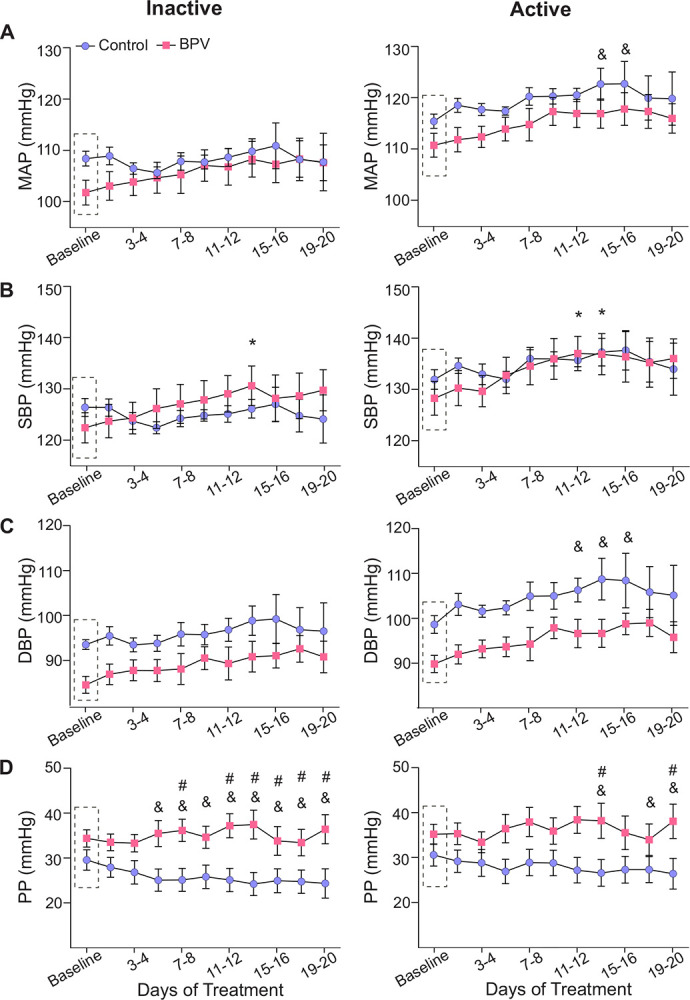
Effects of pulsatile BP on average 12:12 hour cardiovascular variables. **A**, Summary data of two-day averages of MAP, SBP (**B**), DBP (**C**), and PP (**D**) during the inactive (daylight) period (left column) and the active (nighttime) period (right column). Dashed rectangles correspond to the baseline period (~5-day saline infusion). “*” and “&” denote within group comparisons (p < 0.05 vs Baseline) for BPV and control, respectively. “#” denotes p<0.05 between groups comparisons. Two-way ANOVA repeated measures followed by Sidak’s comparison test; n = 12 control mice, n = 8 BPV mice. **BPV**=blood pressure variability, **DBP**=diastolic blood pressure, **MAP**=mean arterial pressure, **PP**=pulse pressure, **SBP**=systolic blood pressure.

Blood pressure presents a circadian rhythm. Thus, cardiovascular variables were assessed during the active (dark) and inactive (light) periods of the mouse 12:12 hr cycle. Similar to the unchanged 24-hour average for MAP, there were minimal changes in the averaged BP corresponding to the daily inactive and active periods for control and BPV mice, Figure 2A–D. Compared to controls, the DBP of the BPV group tended to be lower but not significant, Figure 2C. Starting on day 7 of treatment, BPV (vs control) mice had significantly higher PP during the inactive period, Figure 2D. These data suggest a compensatory mechanism for maintaining MAP within physiological ranges, albeit elevated PP in BPV mice.

### Pulsatile Ang II infusion induced high BPV

Having achieved minimal changes in the 24-hour averaged MAP, yet with prominent BP fluctuations, we quantified blood pressure variability (BPV) over time. Two indices of BPV were used: the average real variability (ARV), defined as the absolute difference between consecutive BP measurements, and the coefficient of variation (CV), defined as the ratio of the standard deviation to mean BP. A significant increase in BPV was observed over time in the BPV group compared to baseline, without any differences in normal 24-hour BP averages. During the active period, ARV was significantly increased by day 3 and persisted through the protocol with 16.4 ± 0.66 mmHg (P=0.04) for SBP and 8.12 ± 0.71 mmHg (P=0.04) for PP, Table 1A. Similarly, during the inactive period, ARV was significantly increased by day 3 and persisted with 13.4 ± 0.33 mmHg (P<0.01) for MAP, 17.3 ± 0.48 mmHg (P=0.02) for SBP, 11.3 ± 0.47 mmHg (P<0.01) for DBP, and 7.9 ± 0.55 mmHg (P=0.04) for PP (Table 1B) with SBP as the main variable driving BPV increases in this model. BPV indices using CV, determined from the average hourly BP over the course 24 hours, were significantly increased from day 1 of treatment for all cardiovascular variables during both the inactive and active periods, except for DBP of the active period, which increased on day 3, **Supplement Table 1**.

### Autonomic function in BPV mice

**Figure 3.**
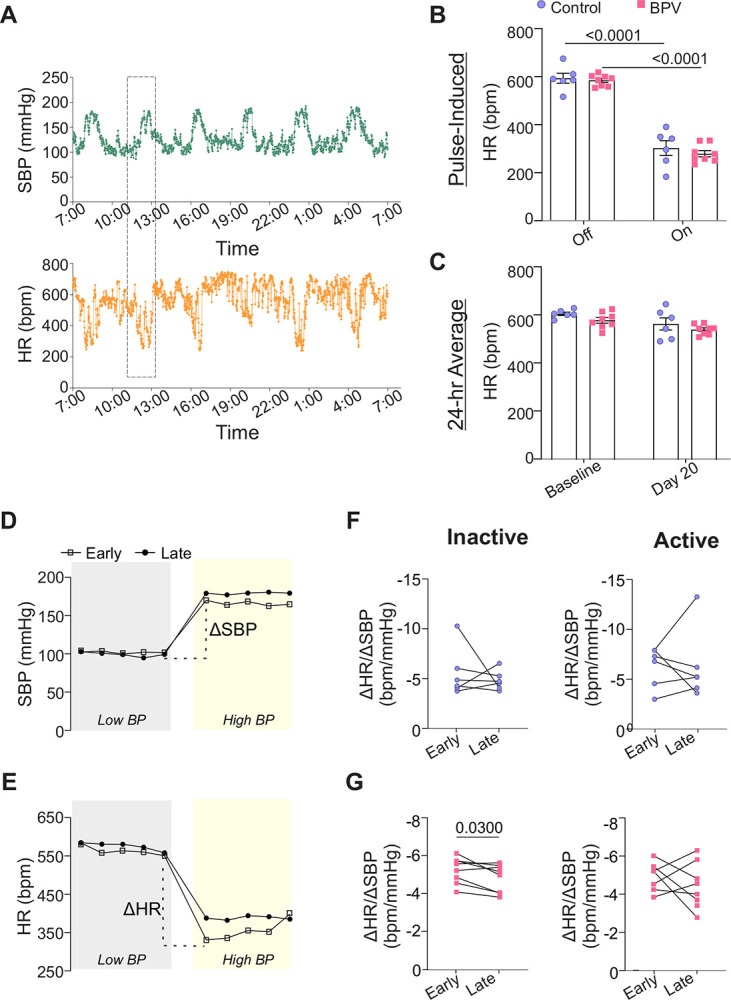
Chronic BPV suppresses bradycardic reflex. **A**, Representative raw traces of the 24-hour SBP (top) and HR (bottom) during intermittent Ang II infusions. **B**, Five-minute average HR when the pump is *Off* or *On* (infusing Ang II) for control and BPV groups extracted from the inactive (daytime) period. **C**, Twenty-four-hour average HR during ~5-day saline infusion (Baseline), and on day 20 of treatment (Day 20) for control and BPV mice. **D**, Representative (5 minute) raw trace of SBP and HR (**E**) from one mouse while the pump is *off* (Low BP, grey) and then *on* (High BP, yellow), extracted from the inactive period. The Early and Late periods correspond to days 3–5 and days 23–25 of the treatment phase, respectively; dashed line denotes delta (Δ) between the Low and High SBP (D) and Low and High HR (E). **F**, Summary data of the bradycardic gain of control and BPV mice (**G**) during the inactive (left) and active period (right). Two-way ANOVA repeated measures followed by Sidak’s multiple comparisons test (B-C, n = 6 control mice, n= 8 BPV mice). Paired t-test and Wilcoxon (F, n = 6 control mice) (G, n = 8 BPV mice). **bpm** = beats per minute, **BPV** = blood pressure variability, **HR** = heart rate, **SBP** = systolic blood pressure.

To compare pressure-induced bradycardic responses during an early and later treatment stage, a cohort of control mice was infused with Ang II during days 3–5 (early phase) (**Supplemental Figure 3**) and on days 21–25 (late phase) of the protocol design. Ang II evoked significant increases in SBP in both the control and BPV groups; these were accompanied by a pronounced bradycardic response, Figure 3A. For a single infusion-evoked BP peak, changes in HR were Δ−291±34 bpm (P<0.0001) and Δ−304±17 bpm (P<0.0001) for control and BPV mice, respectively, Figure 3B. While each Ang II infusion evoked significant bradycardic responses, the 24-hour HR average was not significantly altered, corresponding to 612±7 and 585±12 bpm for baseline and 570±25 and 546±8 bpm at day 20, for control and BPV mice, respectively, Figure 3C.

To determine if the baroreflex response was affected by chronic BP fluctuations, we measured the bradycardic gain expressed as the delta HR over the delta SBP (ΔHR/ΔSBP), focusing on within-group differences, Figure 3D–G. Comparisons were made using five-minute averages (Ang II-evoked BP pulse occurring during the active and inactive period) and between the early and late treatment period. While there were no differences in the control group, the BPV group showed a significant (P<0.03) reduction in the bradycardic gain during the inactive period over time, Figure 3G. These data suggest that chronic (~25 days) BPV decreases bradycardic responses, supporting suppressed autonomic function.

### Chronic BPV enhanced parenchymal arteriole myogenic responses

**Figure 4.**
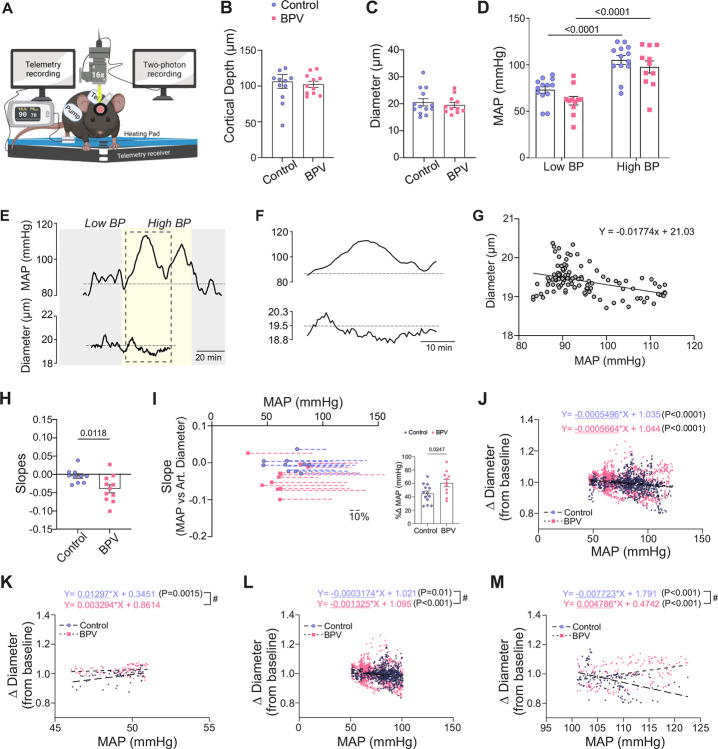
Enhanced myogenic responses in parenchymal arterioles of BPV mice. **A**, Illustration of the experimental set-up including a portable telemetry system for blood pressure recordings simultaneously with 2P imaging recordings of parenchymal arteriole diameter. **B**, Summary data of the average depth of acquisition below the surface of the brain where imaging was conducted. **C**, Summary data of the average baseline diameters of parenchymal arterioles imaged. **D**, Summary data of the average MAP when the infusion pump was *off*, low blood pressure (Low BP) range, and when the infusion pump was *on*, high blood pressure (High BP) range. **E**, Representative raw trace (from one mouse) of MAP (top) and parenchymal arteriole diameter (bottom). **F**, Expanded data corresponding to dashed square in (F), during the Ang II evoked BP changes. **G**, Representative scatter plot of MAP vs parenchymal arteriole diameter during an imaging run. **H**, Summary data of the slopes of the MAP-diameter linear regression, as shown in (G). **I**, The minimum MAP during an acquisition run vs the slope in (H); dashed lines correspond to the percent Δ change in MAP from the minimum MAP value of the run. Summary of percent Δ change in MAP from the minimum MAP shown in insert. **J**, Scatter plot of MAP vs delta diameter from baseline pressure (~70 mmHg) with a pressure range 46–122 mmHg, ≤50 mmHg (**K)**, 51–100 mmHg (**L**), ≥101 mmHg (**M**). Unpaired t-test and Mann-Whitney test (B-C, H-I, n = 13 runs/9 control mice, 11 runs/8 BPV mice). Two-way ANOVA repeated measures followed by Sidak’s multiple comparison test (D, n = 13 runs of 9 control mice, 11 runs of 8 BPV mice). Simple linear regression and “#” denotes p < 0.05 between group (J-M, n = 12 runs/9 control mice, n= 8 runs/7 BPV mice). **Art. Diameter** = parenchymal arteriole diameter, **BPV** = blood pressure variability, **MAP** = mean arterial pressure

**Figure 5.**
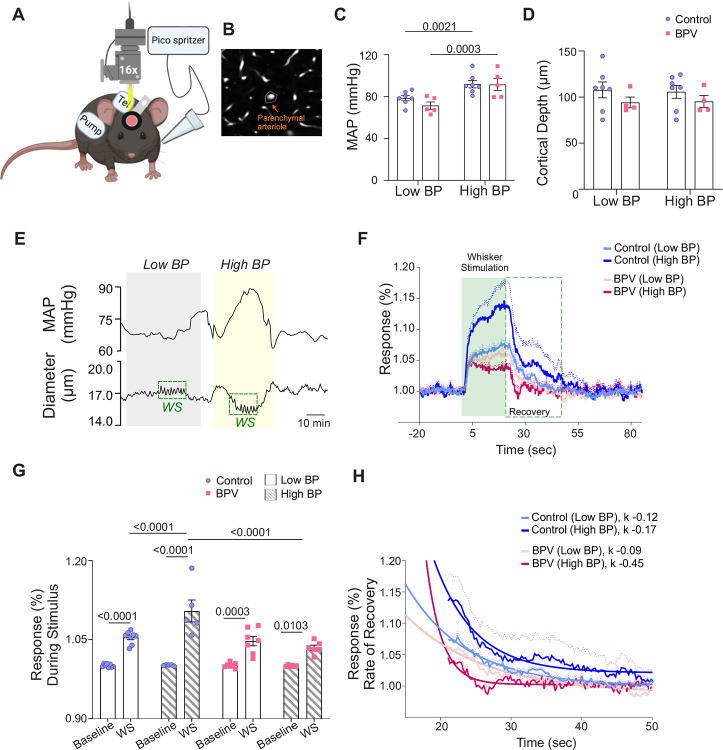
Suppressed neurovascular responses in parenchymal arterioles of BPV during low and high blood pressure periods. **A**, Illustration of the experimental setup in which a picospritzer delivered a puff of air for whisker stimulation (WS) at a rate of 10 Hz for 20 seconds. **B**, Representative image showing the mask (outlined) used to track parenchymal arteriole diameter changes. **C**, Summarized data of average MAP during periods when pump is *off* (Low BP) and *on* (High BP). **D**, Summary data of averaged depth below the surface of the brain where imaging was conducted. **E**, Representative raw trace (from one mouse) of MAP (top) and parenchymal arteriole diameter (bottom) during low and high blood pressure periods; green traces correspond to the WS period response and “a” denotes the 30 seconds pre-stimulus diameter, while “b” denotes the 30 seconds post-stimulus diameter, summarized in (**F**). **G**, Normalized averaged arteriole diameter traces with the corresponding error bars as dashed lines during the WS response shown as % change from baseline (20 seconds before stimulus), 20 seconds during the stimulus (green shade), and 64 seconds post-stimulus. **H**, Summarized data of stimulus-induced arteriole response (green shade in G). **I**, Summary data of recovery time with rate of decay “ƙ” corresponding to 30 seconds post-stimulus outlined in the green dashed square in panel G. Two-way ANOVA repeated measures followed by Sidak’s multiple comparisons test (C-D, n = 7 control mice, 5 BPV mice) (F, n = 5–8 control mice, 6–7 BPV mice) and (H, n = 5–8 control mice, 6–7 BPV mice). One phase exponential decay nonlinear fit (I, n = 5–8 control mice, 6–7 BPV mice). **BPV** = blood pressure variability, ***k*** = rate of decay, **MAP** = mean arterial pressure, **WS** = whisker stimulation

CBF regulation involves complex processes that maintain the metabolic needs of working neurons. These include mechanisms active at baseline (e.g., cerebral autoregulation^16^ and endothelial cell-mediated signaling^32^) and those involved in activity-mediated increases in CBF or neurovascular coupling (NVC)^14^. To assess if BPV impacts the functional integrity of the neurovascular complex, we measured parenchymal arteriole responses to acute BP increases and determined if BP itself altered the sensory-evoked arteriole response. For these studies, *in vivo* two-photon imaging was combined with BP measurements via telemetry, Figure 4 and 5.

To determine the impact of acute BP changes on the microcirculation, we simultaneously tracked parenchymal arteriole diameter changes before pump infusion onset (Low BP), and during Ang II infusions (High BP), Figure 4E. For these experiments, the pump reservoir for the control group was switched from saline to Ang II. Imaging was conducted at similar cortical depths (~ layer 1–2 border of the somatosensory cortex) corresponding to 106±9.6 μm and 102±4.3 μm for control and BPV mice, respectively, Figure 4B. Arteriole diameters measured at baseline (Low BP) were comparable between groups, corresponding to 20±1.3 μm and 19±1.0 μm for controls and BPV mice, respectively, Figure 4C. Upon mild sedation resting BP dropped by −23±3.6 mmHg and −31±4.4 mmHg for the control and BPV group, respectively (not shown). However, BP values remained within the putative cerebral autoregulation plateau range throughout two-photon imaging sessions and were comparable between groups. During the pump infusion period (High BP), the BP increased from 72±3.6 to 103±4.7 mmHg for controls (P<0.0001) and from 60±4.4 to 96±6.4 mmHg for BPV mice (P<0.0001), Figure 4D.

To quantify *in vivo* pressure-evoked diameter changes (myogenic constrictions), we compared the slopes extracted from the linear regression corresponding to the MAP vs parenchymal arteriole diameter measured throughout the acquisition session, Figure 4E–G. As determined by a steeper negative slope, the reactivity of parenchymal arterioles to BP increases in BPV mice (vs control) was significantly larger (P=0.0118), Figure 4H. However, because under sedation, BPV mice showed lower baseline BP, albeit not significant (P=0.18, Figure 4D), we considered if differences in the pressure ranged (i.e. change in pressure between low and high BP intervals) might explain the steeper slopes found in BPV mice. Figure 4I shows the relationship between the lowest BP measured during the acquisition period and the derived slope from the MAP-diameter linear regression analysis. Here, dashed lines represent the percent change in MAP from the starting minimal BP. The BPV mice showed a significantly greater (P=0.0247) percent change in BP (%Δ61±5.4 mmHg vs %Δ45±3.8 mmHg for control mice, Figure 4I, insert). However, the average BP range for controls was still sufficient for computing a correlation between BP and diameter, thus supporting differences in the myogenic response and not a consequence of limited BP range in control animals.

The static cerebral autoregulation curve (CA) described by Lassen in 1959 is characterized by a plateau phase in the MAP-CBF relationship, sandwiched between a lower and an upper limit range^33^. The shape of the curve has been challenged^34^; evidence supports a narrower plateau range and a potential dilatory phase as BP decreases or increases from the plateau range^35^. We determined the pressure-diameter relationship of the whole cohort of mice. As shown in Figure 4J, parenchymal arterioles from control and BPV mice showed significant increases in tone (P<0.0001) with increasing pressures from ~50 to 125 mmHg. When we compared the slopes of the segmented regression lines to apparent lower and higher limits of autoregulation and the plateau range^35^, our data supports a significant decrease in arteriole diameter (P<0.0015) in control mice at pressures <50 mmHg (Figure 4K). At a putative CA plateau range for these arterioles^35^ 51–100 mmHg both groups showed significant constrictions (P<0.001 for controls and P<0.0001 for BPV arterioles) (Figure 4L), with steeper slopes for the BPV group (P<0.05). At pressures >100 mmHg, arterioles from BPV mice showed significant dilations (P<0.001) while those from control mice showed significant constrictions (P<0.001), Figure 4M. Together, these data support changes in the CA curve of BPV mice corresponding to a shifted plateau range, with enhanced myogenic constrictions at comparable pressure, but significant dilations at greater pressures where controls arterioles still hold tone.

### Impaired NVC in high BPV mice

Because mechanisms underlying baseline CBF regulation differ from those evoked during the functional hyperemia response, we assessed whether chronic BPV affected functional-evoked outputs or neurovascular coupling (NVC). Functional hyperemia was assessed using two-photon imaging and evoked using air puffs to stimulate the mouse whiskers. Blood pressure measurements were simultaneously measured via telemetry, Figure 5A. While sensory-evoked vascular responses are commonly used as a proxy for neuronal activation and function^18, 36^, few studies have considered (or reported) BP during these *in vivo* protocols. Thus, we asked if chronic and acute BP fluctuations alter the NVC response at the level of parenchymal arterioles, Figure 5B. NVC responses were compared during low and high BP periods corresponding to pumps being *off* or *on*, respectively, Figure 5E.

The average baseline (low BP) MAP was 78±1.9 mmHg and 71.0±3.4 for the control and BPV group, respectively, Figure 5C. Imaging was conducted at similar cortical depths within ~ layers 1 & 2 of the somatosensory cortex, corresponding to 103±8.0 μm and 96±3.0 μm for control and BPV arterioles, respectively, Figure 5D. To compare NVC responses at low and high BP, the pump reservoir for the control group was switched from saline to Ang II. Ang II infusion evoked significant (and comparable) increases in MAP, corresponding to 91±3.0 mmHg for controls and 92±5.6 mmHg for BPV mice, Figure 5C. Whisker stimulation caused significant dilations (vs. baseline) in all groups, regardless of MAP, Figure 5F–G. However, at higher BP, the magnitude of the NVC response in control mice was greater (P<0.0001, high BP vs low BP). Notably, this pressure-dependent response was abrogated (P=0.60) in the BPV group, Figure 5G. A closer look at the post-stimulus recovery phase showed a faster recovery rate (k = −0.45) for parenchymal arterioles of BPV mice during high BP periods compared to controls (P<0.0001), Figure 5H. These data revealed an acute pressure-dependent effect in control mice, with higher pressures resulting in greater sensory-evoked dilations. Additionally, the data shows that chronic BP fluctuations abrogated the pressure-dependent effects observed in control mice.

### Cognitive decline in high BPV mice

**Figure 6.**
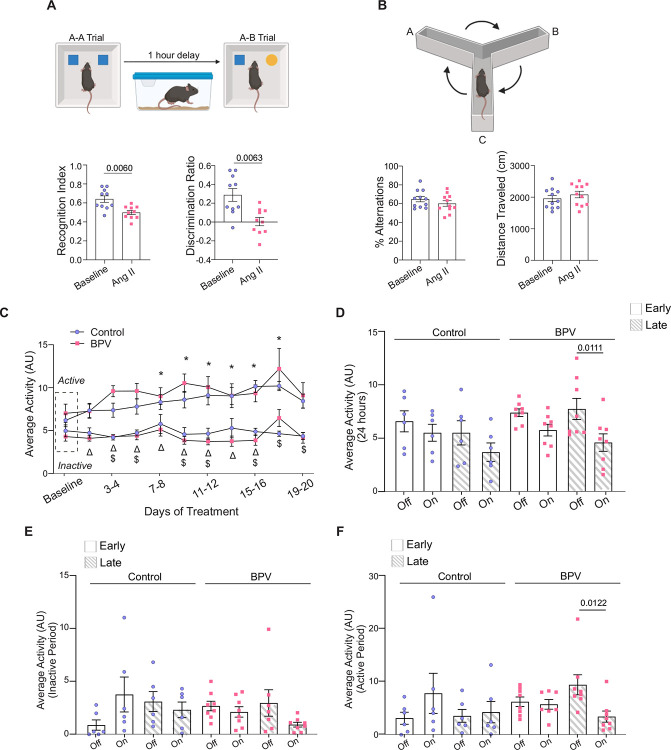
Behavior and altered cognitive function of BPV mice. **A**, Illustration of the novel object recognition test with a 1-hour delay between the A-A and A-B trials (top) with corresponding summarized data of mice recognition index and discriminatory index during the saline infusion period (baseline) and following 25 days of pulsatile Ang II infusion (Ang II). **B**, Diagram of 10-minute spontaneous Y-maze experimental preparation (top) and the summary of % alternation during baseline and 25 days of Ang II (bottom). **C**, Summary data of two-day 24-hour averages of activity throughout treatment for active and inactive periods. Baseline (~5 day saline infusion) is marked by the dashed rectangle. **D**, Summary data showing 24-hour averaged activity when the pump is *on* or *off* during days 3–5 (Early) and days 23–25 (Late) of treatment. Summary of activity when the pump is *on* and *off* during the Early and Late phases of treatment for the inactive cycle (**E**) and active cycle (**F**). Paired t-test (A, n = 10 BPV mice), (B, n = 11 BPV mice). Two-way ANOVA repeated measures followed by Tukey’s comparison test (C, n = 11 control mice, 8 BPV mice) (D-F, n = 6 control mice, 8 BPV mice). “*” denotes p < 0.05 vs Baseline for control, active period. “$” and “Δ” denote p<0.05 (active vs inactive period) for control and BPV, respectively. **AU**=arbitrary units, **BPV**=blood pressure variability

Given impaired neurovascular outputs and the established association between high BPV and cognitive decline^37^, we used the NOR and Y-maze tests to assess cognitive performance. Mice were subjected to behavioral testing during the saline-infused period and following 25 days of BPV (via Ang II infusions). Using NOR, a significant decrease in the recognition index (P=0.006) and discriminatory index (P=0.006) was observed (Figure 6A), supporting impaired recognition memory. No differences were observed in the short-term spatial working memory assessed via Y-maze alternation (P=0.25), Figure 6B.

Mice activity levels were evaluated as another aspect of behavior. Throughout the study, activity remained high during the active vs inactive periods (P=0.049 for control, P=0.0287 for BPV), Figure 6C. In addition, there were no differences in the average activity level between groups, regardless of pump *on-off* status (24-hour, 12 hr-inactive, or 12 hr-active) or treatment regimen Figure 6D–F. However, following 23–25 days of treatment (Late), the activity of BPV mice significantly decreased when the pump was *on*. This observation was evident in the data corresponding to 24-hour averages (P=0.0111, Figure 6D), as well as during the active period of the 12-hour activity averages (pump *on* vs *off*, P=0.0122, Figure 6F). These data support that mice subjected to chronic BPV become less active when the BP increases or the pump status is *on*. Collectively, while the infusion protocol does not disrupt BP-related circadian rhythms, 25 days of chronic BPV compromised recognition memory and mice activity.

## DISCUSSION

BPV has emerged as a risk factor for cognitive decline, but its impact on brain function remains poorly understood. Here, we introduce a novel murine model of BPV using pulsatile BP increases induced via Ang II infusions without hypertension. Using *in vivo* two-photon imaging, we provide evidence of altered vascular function in mice subjected to chronic BP fluctuations. In BPV mice, parenchymal arterioles of <30 μm showed enhanced myogenic constrictions. In addition, chronic BPV impaired neurovascular outputs and cognitive function. Notably, the NVC response in control mice was pressure-dependent, with greater magnitudes when the arterial BP was increased. This pressure-dependent effect was lost in mice subjected to chronic BPV. These data support the notion that chronic BPV targets a broad range of physiological processes (e.g., bradycardic reflex, myogenic reactivity, neurovascular coupling) and is a critical risk factor for brain health.

Our model emphasizes the importance of BPV assessment regardless of averaged BP values. Retrospective studies in primary care patients reveal BPV occurrences in both hypertensive and non-hypertensive adults^38^, placing BPV as a critical modifiable risk factor, often overlooked by standard in-office single BP measurements. The significantly higher 24-hour averaged PP in the BPV group (**Supplemental Figure 2B**) makes PP the most sensitive BP-related variable in our model. The BPV group (vs controls) showed sustained lower DBP (Figure 2C) and higher PP during the inactive period (Figure 2D), suggestive of a compensatory mechanism, which maintained the 24-hour MAP within physiological values. However, other factors preventing the onset of hypertension cannot be ruled out. To this end, experiments from control vs experimental mice were not equally conducted in the same season raising the possibility for a seasonal effect^39, 40^.

The autonomic nervous system (ANS) tightly regulates BP via changes in heart rate, systemic vascular resistance, and stroke volume. Notably, the baroreflex maintains cerebral perfusion pressure^41^. Acute increases in BP, causes arterial baroreceptors to inhibit sympathetic outflow and (via enhanced vagal activity) decrease HR and peripheral vascular resistance^41^, consequently decreasing arterial BP. However, to maintain CBF relatively constant, baroreflex-mediated inhibition of sympathetic outflow and arteriole dilation is countered by the intrinsic properties of the cerebral vasculature, which constricts in response to increases in arterial BP. Dysregulation of these dynamic processes can put the brain at risk for both hypoperfusion and hyperperfusion. Impaired baroreflex sensitivity correlates with aging^42^ and mild cognitive impairments^43^. In our model, chronic BPV significantly blunted the baroreflex response, suggestive of ANS dysregulation. Previous reports have shown that chronic Ang II infusion suppresses and resets baroreceptor sensitivity independently of the pressure effect, but the resetting partially reverses within 30 minutes post-Ang II infusions^44^. In contrast to commonly used approaches of sustained Ang II infusion (osmotic pump models), our protocol delivers Ang II intermittently at 3- or 4-hour intervals. Thus, we speculate that this interval prevents desensitization or Ang II receptor internalization, another well-established phenomenon arising from sustained infusions^45, 46^. In addition, the same Ang II dose evoked comparable BP increases at the start and end of the treatment regimen. The lack of differences in the bradycardic gain during the active period may be attributed to activity-mediated HR increases. Thus, our results support diminished baroreflex responses, which aligns with previous reports showing an association between high BPV and autonomic dysregulation^47^. The cellular mechanisms and targets are, however, poorly understood.

As mentioned above, cerebral autoregulation protects against fluctuations in cerebral perfusion pressure^22^. It is expected that vessels upstream from cerebral capillaries (e.g., small pial arterioles and arterioles^33, 35, 48^, larger vessels^49–51^) buffer arterial pressure changes, sparing the brain from large CBF variations. Here, we show *in vivo* myogenic responses of parenchymal arterioles deep into the brain. Notably, responses were enhanced in the BPV group. While *ex vivo* studies have confirmed prominent myogenic responses in parenchymal arterioles^21, 52–54^, chronic BPV-evoked changes to the reactivity of parenchymal arterioles to pressure suggest dominance of vasoconstrictive mechanisms driving smooth muscle cells to a more depolarized state (e.g., increased expression of voltage-dependent calcium channel (VDCC) or decreased K^+^ currents^55^) or vascular remodeling in upstream vessels reducing pressure-buffering mechanisms to the microcirculation^56^. Cipolla et al., showed that compared to middle cerebral arteries, parenchymal arterioles were 30-fold more sensitive to the VDCC blocker nifedipine^57^. Importantly, the expression of VDCCs increases in hypertension^58–64^ and long-acting calcium channel blockers (i.e., amlodipine), are among effective treatments for BPV^65, 66^. A limitation of our study was that under the same experimental conditions, mild sedation induced a lower baseline BP (pumps *off*) in BPV mice compared to controls. Because the peak BP during the infusion period was comparable between groups, the lower BP baseline broadens the range used to determine the slope of the myogenic response. To address this limitation, we compared the pressure-arteriole diameter change of the mice cohort. Here, we observed notable differences in the shape of the curve supporting enhanced myogenic constrictions at comparable pressure ranges. In addition, we observed a shift in the plateau phase with prominent dilations occurring at pressures >100 mmHg where control mice arterioles still held tone. This data supports variations in the cerebral autoregulation curve of BVP mice, which may compromise perfusion. Future studies addressing the impact of BPV on capillary perfusion or red blood cell velocity may better explain the link between BPV and brain pathology, such as microbleeds.

Chronic BPV significantly blunted the NVC response to whisker stimulation. Notably, control mice showed a pressure-dependent effect in the magnitude of the NVC response, which was abrogated in BPV mice. One possible explanation is that the greater NVC response in control mice arises from higher arteriole tone and a more efficient dilatory response to vasoactive signals released during the stimulus. On the other hand, the observation that arterioles from BVP mice, when subjected to higher pressures, showed faster recovery in diameter post-stimulus is also suggestive of a dominant vasoconstrictive state^67^. Another possibility is that the blunted NVC response is of neuronal origin, such as a decreased in activity or in the release of vasodilatory signals. It would be important to assess the impact of high BPV on the neurovascular complex under awake conditions, with the caveat that activity and arousal^68, 69^, among other factors, can increase BP and thus interfere with the pressure-dependence effect of neurovascular outputs.

Along with a blunted NVC response, our study shows that chronic BPV leads to cognitive decline as measured by a decrease in the recognition index in the NOR test. While no differences were observed in Y-maze alternations, these could be attributed to the age of the mice (14 months-old) which may already exhibit cognitive deficits^70^. These were evident when assessing the time-delay between the A-A and A-B trials in the NOR test, which lead us to use a shorter time window between testing.

### Conclusions

In conclusion, our study reveals that high BPV disrupts cellular communication at the neurovascular complex, contributing to cognitive decline. Notably, our model achieved large BP fluctuations while preventing the onset of hypertension, providing evidence for the causal role of high BPV on poor neurovascular outputs. These findings advocate for including BPV assessment as a critical screening and diagnostic tool alongside standard average BP measurements, addressing cardiovascular and neurovascular risks. The adaptable model developed in this study holds promise for investigating the impact of BPV on brain function.

## Figures and Tables

**Table 1.** Ang II-induced increases in blood pressure variability (BPV).

A								
			Active Period ARV mmHg ± SEM					
	MAP		SBP		DBP		PP	
Days	Control	BPV	Control	BPV	Control	BPV	Control	BPV
*Baseline*	*10.16 ± 0.79*	*10.94 ± 0.55*	*12.47 ± 0.71*	*13.13 ± 0.66*	*9.84 ± 0.62*	*9.04 ± 0.54*	*4.21 ± 0.34*	*5.47 ± 0.46*
1–2	11.66 ± 0.78	10.99 ± 0.35	14.73 ± 1.02	14.56 ± 0.44	11.18 ± 1.04	9.29 ± 0.41	5.39 ± 0.49	7.67 ± 0.69
3–4	11.83 ± 0.66	12.51 ± 0.43	14.77 ± 0.80	16.40 ± 0.66 *	10.82 ± 0.69	10.35 ± 0.45	5.28 ± 0.53	8.12 ± 0.71 *
5–6	11.33 ± 0.60	12.80 ± 0.39	14.51 ± 1.12	16.44 ± 0.53 *	11.30 ± 1.22	10.54 ± 0.45	5.03 ± 0.52	7.99 ± 0.69 *^#^
7–8	11.96 ± 0.84	12.85 ± 0.41 *	15.85 ± 1.52	16.73 ± 0.43 *	12.44 ± 1.63	10.61 ± 0.55 *	5.21 ± 0.53	8.34 ± 0.74 *^#^
9–10	11.88 ± 0.83	12.84 ± 0.40	14.66 ± 1.13	16.37 ± 0.72 *	11.81 ± 1.19	10.82 ± 0.24	4.95 ± 0.50	7.86 ± 0.65 *^#^
11–12	12.75 ± 1.01	12.94 ± 0.76 *	15.80 ± 1.23	16.87 ± 0.70 *	12.68 ± 1.26	10.59 ± 0.81	5.12 ± 0.42	8.25 ± 0.78 ^#^
13–14	11.80 ± 1.01	12.90 ± 0.42 *	15.16 ± 1.38	16.80 ± 0.52 *	11.98 ± 1.39	10.40 ± 0.68 *	5.16 ± 0.48	8.45 ± 0.83
15–16	12.33 ± 0.72	13.91 ± 1.02	15.11 ± 0.85	17.50 ± 0.87 *	11.73 ± 0.97	11.59 ± 1.14	5.34 ± 0.44	8.25 ± 0.73 *
17–18	12.17 ± 0.99	14.06 ± 0.92 *	15.76 ± 1.34	17.12 ± 0.77 *	12.31 ± 1.17	11.95 ± 1.19	5.53 ± 0.67	7.60 ± 0.77
19–20	11.51 ± 0.74	12.75 ± 0.92	15.27 ± 1.02	16.57 ± 0.94	11.49 ± 1.16	10.48 ± 1.23	6.09 ± 0.72	8.44 ± 1.15
B								
			Inactive Period ARV mmHg ± SEM					
	MAP		SBP		DBP		PP	
Days	Control	BPV	Control	BPV	Control	BPV	Control	BPV
*Baseline*	*9.44 ± 0.75*	*11.29 ± 0.54*	*11.64 ± 0.84*	*13.28 ± 0.70*	*9.34 ± 0.63*	*9.48 ± 0.45*	*4.18 ± 0.36*	*5.04 ± 0.45*
1–2	10.73 ± 0.66	11.40 ± 0.69	13.61 ± 0.76	14.69 ± 0.77	10.33 ± 0.79	9.76 ± 0.68	4.94 ± 0.31	7.09 ± 0.55
3–4	11.35 ± 0.84	13.38 ± 0.33 *	13.89 ± 0.68	17.27 ± 0.48 *#	10.66 ± 0.77	11.29 ± 0.47 *	4.83 ± 0.40	7.90 ± 0.55 *#
5–6	11.65 ± 0.63	13.63 ± 0.78 *	14.69 ± 0.80	17.57 ± 0.78 *	11.07 ± 0.91	11.34 ± 0.86	5.17 ± 0.44	8.23 ± 0.69 *#
7–8	11.29 ± 0.67	13.73 ± 0.50 *	14.28 ± 0.99	17.44 ± 0.53 *	11.25 ± 1.05	11.61 ± 0.67 *	4.96 ± 0.44	8.45 ± 0.89
9–10	12.78 ± 1.60	14.77 ± 0.66 *	15.76 ± 1.81	18.52 ± 0.72 *	12.67 ± 1.50	12.59 ± 0.64 *	5.40 ± 0.41	8.16 ± 0.61 *#
11–12	11.93 ± 1.09	14.53 ± 0.57 *	14.78 ± 1.20	18.65 ± 0.62 *	12.23 ± 1.46	12.09 ± 0.74 *	5.23 ± 0.38	8.50 ± 0.88
13–14	12.23 ± 0.97	13.96 ± 0.74 *	15.32 ± 1.12	17.65 ± 0.53 *	12.29 ± 1.28	11.56 ± 0.92	5.11 ± 0.44	8.16 ± 0.74 *#
15–16	12.31 ± 1.11	15.04 ± 0.87 *	15.17 ± 1.21	18.85 ± 0.87 *	12.39 ± 1.22	12.66 ± 1.07 *	4.86 ± 0.36	8.54 ± 1.06 *
17–18	12.07 ± 0.91	15.61 ± 1.36	14.79 ± 1.02	19.39 ± 1.09 *	11.74 ± 1.02	13.18 ± 1.67	5.07 ± 0.44	8.46 ± 1.03
19–20	11.82 ± 0.79	14.72 ± 1.00 *	15.56 ± 1.07	18.96 ± 0.96 *	11.65 ± 0.98	12.19 ± 1.25	6.00 ± 0.41	9.28 ± 1.33

**A-B**, Summary data of calculated two-day average real variability of MAP, SBP, DBP, and PP during the active period (**A**) and the inactive period (**B**). Baseline corresponds to (~5-day saline infusion) and “*” denotes within group comparisons (p < 0.05 vs Baseline) and “#” denotes p < 0.05 between groups comparisons. Two-way ANOVA repeated measures followed by Dunnett’s comparison test; n = 11 control mice, n = 8 BPV mice.

**ARV**=average real variability, **BPV**=blood pressure variability, **DBP**=diastolic blood pressure, **MAP**= mean arterial pressure, **PP**=pulse pressure, **SBP**=systolic blood pressure.
