# Supplementary figures and images for: Blood pressure variability compromises vascular function in middle-aged mice
Source: bioRxiv. 2025 Jul 2:2024.10.21.619509. Originally published 2024 Oct 24. Preprint. [Version 2] doi: 10.1101/2024.10.21.619509 (PMC11526967; doi:10.1101/2024.10.21.619509)

# Suppl. Fig 1

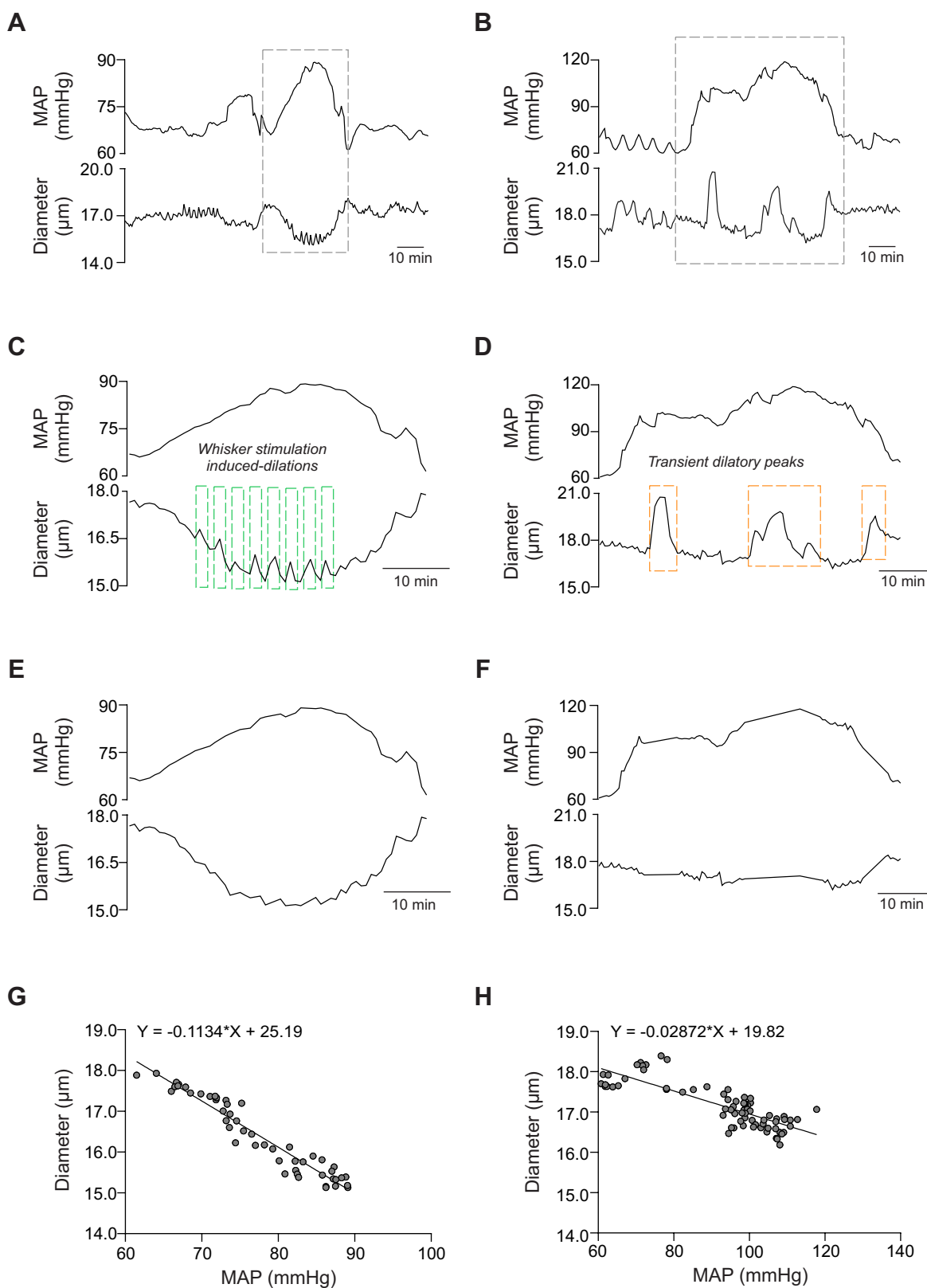

Supplement: Supplement 1 — Supplemental Figure 1. Exclusion criteria for myogenic response analysis. A-B, Representative raw traces (from one mouse) of MAP (top) and parenchymal arteriole diameter (bottom) showing WS-evoked dilations (A) and random dilatory events (B). Grey dashed rectangles outline the period when the pump is on and BP transitions to higher values (Ang II infusion). C-D, Expanded data corresponding to the dashed rectangle shown in (A-B), with dilatory responses to WS outlined within green dashed rectangles (C) and transient dilatory events shown within orange dashed rectangles (D). E-F, Representative raw traces of MAP (top) and parenchymal arteriole diameter (bottom) following removal of sensory-evoked dilations (C) or the random dilatory events outlined in (D). G-H, Scatter plot and resultant linear regression line of the filtered MAP vs parenchymal diameter corresponding to (E-F). MAP = mean arterial pressure [file media-1.pdf]

## A 24-hour Averaged BP at Baseline

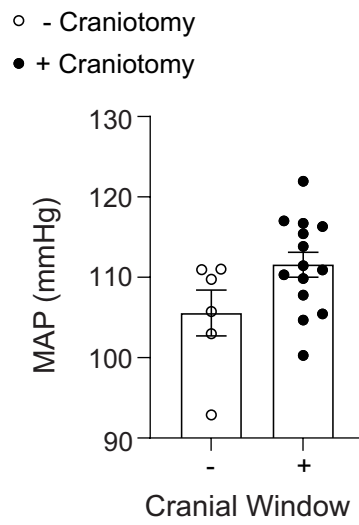

## B 24-hour Averaged BP

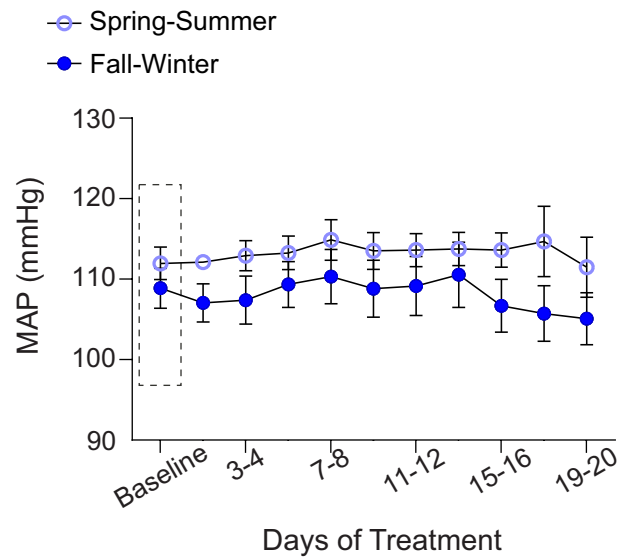

Supplement: Supplement 2 — Supplemental Figure 2. Seasonal effects on mean arterial pressure. A, Summarized data of 24-hr MAP at baseline (5 days saline infusion) in mice with (+) or without (−) a cranial window. B, Summarized data of two-day average MAP at baseline and 20 days of treatment. Spring-Summer corresponds to warmer months (May-September), and Fall-Winter corresponds to colder months (October-April). Mann-Whitney test (A, n = 14 mice with craniotomy, 6 without craniotomy). Two-way ANOVA repeated measures followed by Sidak’s multiple comparisons test (B, n = 11 mice in Spring-Summer, 9 mice in Fall-Winter). MAP = mean arterial pressure [file media-2.pdf]

**A**

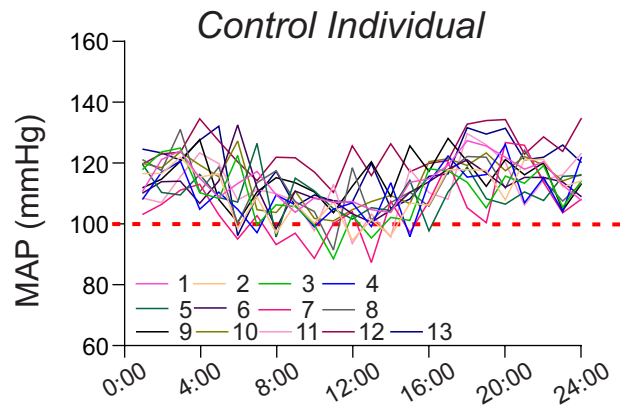

**B**

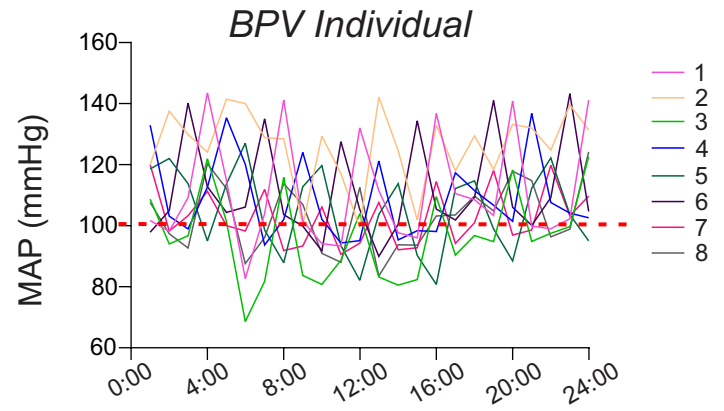

**C**

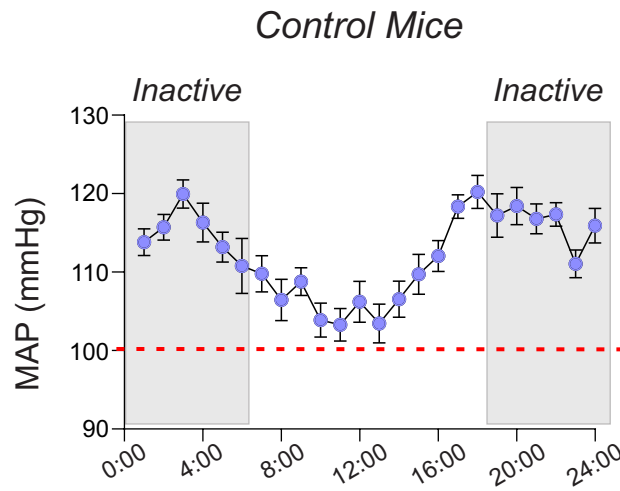

**D**

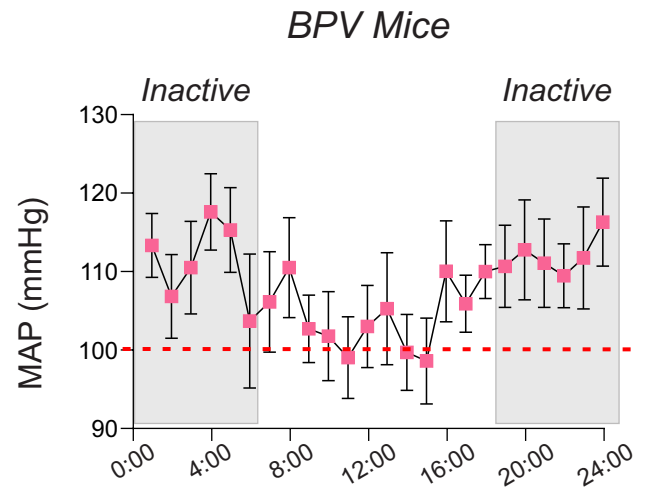

Supplement: Supplement 3 — Supplemental Figure 3. One-hour averages of MAP over 24hrs-Circadian Profile A, Representative raw trace of one-hour average MAP for 24hrs of all control mice and BPV mice (B), with controls summarized in (C) and BPV in (D). Inactive corresponds to daylight hours (6am-6pm). (A-D, n = 13 control mice, n = 8 BPV mice). BPV=blood pressure variability, MAP = mean arterial pressure [file media-3.pdf]

# Suppl. Fig 4

## Infusion-Evoked (Control Group)

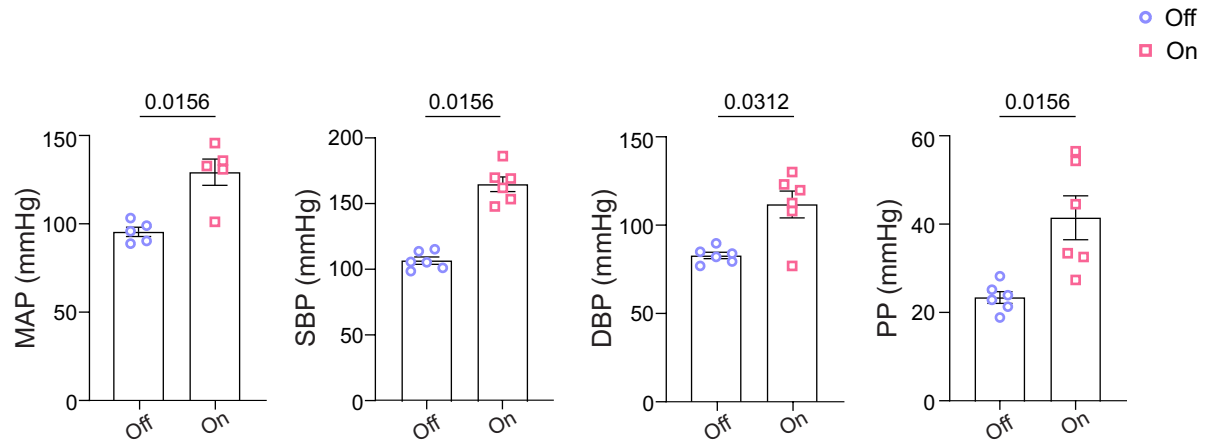

Supplement: Supplement 4 — Supplemental Figure 4. Transient Ang II infusions induced pulsatile blood pressure in control mice. Summary data of averaged BP (5 minutes) while the pump is Off or On and infusing Ang II in controls. Data was extracted from the inactive (daytime) period at days 3-5 of treatment protocol. Paired Wilcoxon test (n = 6 control mice). DBP = diastolic blood pressure, MAP = mean arterial pressure, PP = pulse pressure, SBP = systolic blood pressure. [file media-4.pdf]

## A Low-High BP Transition

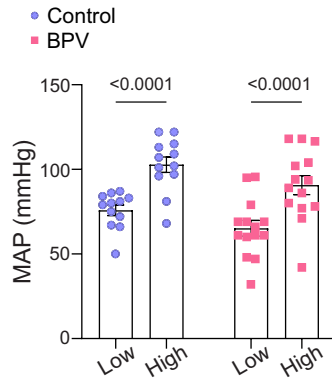

## B

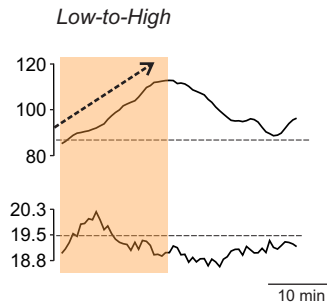

## C

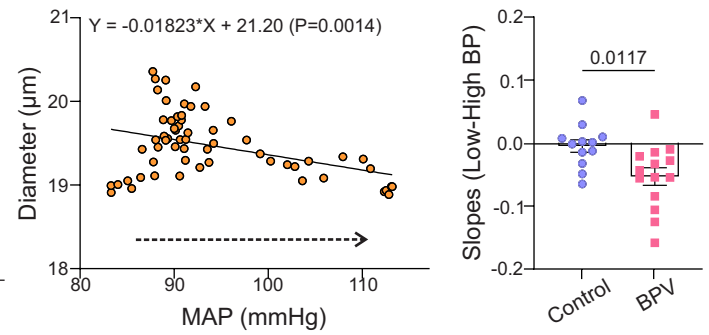

## D High-Low BP Transition

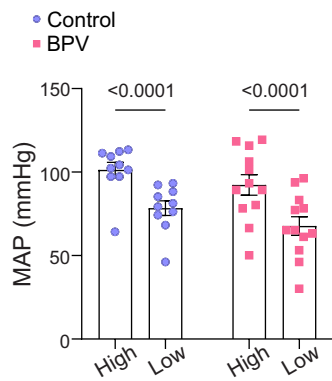

## E

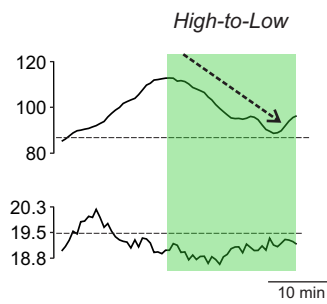

## F

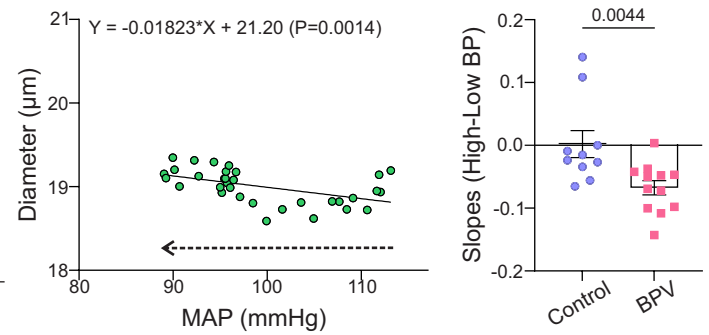

Supplement: Supplement 5 — Supplemental Figure 5. Directional myogenic responses A, Summary data for averaged BP corresponding to the Low-to-High BP transition. B, Representative raw trace showing MAP (top) and parenchymal arteriole diameter (bottom) during Low-to-High MAP transition shaded in orange (left) and the corresponding representative scatter plot of MAP vs parenchymal arteriole diameter (right). C, Summary of MAP-diameter linear regression slopes corresponding to Low-to-High MAP transition. D, Summary data of the average high and low MAP recorded immediately following cessation of pump infusion and blood pressure transitions from high to low BP (High-to-Low). E, Representative raw trace showing MAP (top) and parenchymal arteriole diameter (bottom) during High-to-Low MAP transition shaded in green (left) and the corresponding representative scatter plot of MAP vs parenchymal arteriole diameter (right). F, Summary of MAP-diameter linear regression slopes corresponding to High-to-Low MAP transition. Two-way ANOVA repeated measures followed by Sidak’s multiple comparisons test (A, n = 12 runs/7 control mice, n = 14 runs/8 BPV mice) (D, n = 10 runs/8 control mice, n = 12 runs/7 BPV mice). Unpaired t-test and Mann-Whitney test (C, n = 12 runs/7 control mice, n = 14 runs/8 BPV mice) (F, n = 10 runs/8 control mice, n = 12 runs/7 BPV mice). BPV=blood pressure variability, MAP = mean arterial pressure [file media-5.pdf]
