# Supplementary material for: Blood pressure variability compromises vascular function in middle-aged mice
Source: bioRxiv. 2025 Jul 2:2024.10.21.619509. Originally published 2024 Oct 24. Preprint. [Version 2] doi: 10.1101/2024.10.21.619509 (PMC11526967; doi:10.1101/2024.10.21.619509)
Supplement: Supplement 6 — Supplemental Table 1. Pulsatile Ang II infusions induced an increase in blood pressure variability (BPV) measured by coefficient of variance. [file media-6.pdf]

Suppl. Table

A

Active Period CV mmHg ± SEM

Days	MAP		SBP		DBP		PP	
	Control	BPV	Control	BPV	Control	BPV	Control	BPV
Baseline	*0.05 ± 0.00*	*0.05 ± 0.00*	*0.05 ± 0.00*	*0.05 ± 0.00*	*0.06 ± 0.00*	*0.05 ± 0.00*	*0.08 ± 0.01*	*0.07 ± 0.01*
1-2	0.06 ± 0.01	0.09 ± 0.01 *	0.06 ± 0.01	0.12 ± 0.01*#	0.06 ± 0.01	0.09 ± 0.01	0.09 ± 0.01	0.22 ± 0.02 *#
3-4	0.05 ± 0.00	0.11 ± 0.01 *#	0.06 ± 0.00	0.13 ± 0.01*#	0.06 ± 0.00	0.11 ± 0.01*#	0.09 ± 0.01	0.20 ± 0.02 *#
5-6	0.05 ± 0.00	0.12 ± 0.01 *#	0.05 ± 0.00	0.13 ± 0.01*#	0.05 ± 0.00	0.12 ± 0.01*#	0.08 ± 0.01	0.20 ± 0.02 *#
7-8	0.05 ± 0.01	0.12 ± 0.01 *#	0.05 ± 0.01	0.14 ± 0.02*#	0.05 ± 0.01	0.12 ± 0.01*#	0.08 ± 0.01	0.22 ± 0.03 *#
9-10	0.05 ± 0.01	0.13 ± 0.01 *#	0.05 ± 0.01	0.15 ± 0.01*#	0.05 ± 0.01	0.14 ± 0.01*#	0.08 ± 0.01	0.21 ± 0.03 *#
11-12	0.05 ± 0.01	0.13 ± 0.01 *#	0.05 ± 0.01	0.15 ± 0.01*#	0.05 ± 0.01	0.12 ± 0.01*#	0.09 ± 0.01	0.22 ± 0.03 *#
13-14	0.05 ± 0.01	0.13 ± 0.01 *#	0.05 ± 0.01	0.15 ± 0.01*#	0.05 ± 0.01	0.13 ± 0.01*#	0.09 ± 0.01	0.24 ± 0.03 *#
15-16	0.04 ± 0.01	0.14 ± 0.01 *#	0.04 ± 0.01	0.16 ± 0.01*#	0.04 ± 0.01	0.13 ± 0.01*#	0.09 ± 0.01	0.26 ± 0.03 *#
17-18	0.05 ± 0.00	0.14 ± 0.01 *#	0.05 ± 0.00	0.16 ± 0.01*#	0.05 ± 0.00	0.14 ± 0.01*#	0.10 ± 0.01	0.26 ± 0.04 *
19-20	0.06 ± 0.01	0.13 ± 0.01 *#	0.06 ± 0.01	0.15 ± 0.01*#	0.05 ± 0.01	0.12 ± 0.01*#	0.12 ± 0.02	0.25 ± 0.02 *#

B

Inactive Period CV mmHg ± SEM

Days	MAP		SBP		DBP		PP	
	Control	BPV	Control	BPV	Control	BPV	Control	BPV
Baseline	*0.06 ± 0.00*	*0.06 ± 0.01*	*0.06 ± 0.00*	*0.06 ± 0.01*	*0.07 ± 0.00*	*0.06 ± 0.01*	*0.11 ± 0.01*	*0.07 ± 0.01*
1-2	0.08 ± 0.01	0.10 ± 0.01*	0.08 ± 0.01	0.12 ± 0.01*	0.08 ± 0.01	0.10 ± 0.01*	0.12 ± 0.03	0.21 ± 0.03*
3-4	0.07 ± 0.00	0.13 ± 0.01*#	0.07 ± 0.01	0.15 ± 0.01*#	0.07 ± 0.01	0.14 ± 0.01*#	0.12 ± 0.02	0.21 ± 0.02*#
5-6	0.07 ± 0.01	0.14 ± 0.01*#	0.07 ± 0.01	0.15 ± 0.01*#	0.07 ± 0.01	0.14 ± 0.02*#	0.12 ± 0.04	0.19 ± 0.02*
7-8	0.07 ± 0.01	0.14 ± 0.01*#	0.08 ± 0.01	0.16 ± 0.01*#	0.07 ± 0.01	0.15 ± 0.01*#	0.11 ± 0.04	0.23 ± 0.04*
9-10	0.07 ± 0.01	0.16 ± 0.01*#	0.07 ± 0.01	0.18 ± 0.02*#	0.07 ± 0.01	0.17 ± 0.01*#	0.10 ± 0.04	0.22 ± 0.03*
11-12	0.07 ± 0.01	0.16 ± 0.01*#	0.07 ± 0.01	0.18 ± 0.01*#	0.08 ± 0.01	0.16 ± 0.01*#	0.12 ± 0.03	0.24 ± 0.04*
13-14	0.08 ± 0.02	0.16 ± 0.01*#	0.08 ± 0.01	0.18 ± 0.01*#	0.08 ± 0.01	0.15 ± 0.02*#	0.12 ± 0.03	0.25 ± 0.04*
15-16	0.07 ± 0.01	0.17 ± 0.01*#	0.07 ± 0.01	0.19 ± 0.01*#	0.07 ± 0.01	0.17 ± 0.01*#	0.13 ± 0.03	0.30 ± 0.05*
17-18	0.08 ± 0.01	0.17 ± 0.00*#	0.08 ± 0.01	0.19 ± 0.01*#	0.08 ± 0.01	0.18 ± 0.01*#	0.14 ± 0.03	0.27 ± 0.04*
19-20	0.07 ± 0.01	0.16 ± 0.01*#	0.08 ± 0.01	0.18 ± 0.01*#	0.08 ± 0.01	0.15 ± 0.01*#	0.14 ± 0.04	0.28 ± 0.04*
